# Self-Determination and Meaningful Work: Exploring Socioeconomic Constraints

**DOI:** 10.3389/fpsyg.2016.00071

**Published:** 2016-02-02

**Authors:** Blake A. Allan, Kelsey L. Autin, Ryan D. Duffy

**Affiliations:** ^1^Department of Educational Studies, Purdue UniversityWest Lafayette, IN, USA; ^2^Department of Psychology, University of FloridaGainesville, FL, USA

**Keywords:** meaningful work, work volition, social class, self-determination theory, psychology of working

## Abstract

This study examined a model of meaningful work among a diverse sample of working adults. From the perspectives of Self-Determination Theory and the Psychology of Working Framework, we tested a structural model with social class and work volition predicting SDT motivation variables, which in turn predicted meaningful work. Partially supporting hypotheses, work volition was positively related to internal regulation and negatively related to amotivation, whereas social class was positively related to external regulation and amotivation. In turn, internal regulation was positively related to meaningful work, whereas external regulation and amotivation were negatively related to meaningful work. Indirect effects from work volition to meaningful work via internal regulation and amotivation were significant, and indirect effects from social class to meaningful work via external regulation and amotivation were significant. This study highlights the important relations between SDT motivation variables and meaningful work, especially the large positive relation between internal regulation and meaningful work. However, results also reveal that work volition and social class may play critical roles in predicting internal regulation, external regulation, and amotivation.

## Introduction

Meaningful work is a fundamental component of well-being (Rosso et al., 2010) that contributes to a more meaningful and fulfilling life (e.g., Baum and Stewart, 1990; Emmons, 2003; Steger and Dik, 2009; Allan et al., 2015). Therefore, understanding causes of meaningful work and helping people manage and construct more meaningful work is an important focus of scholarship and career interventions, both on individual and organizational levels (Guichard, 2013). Meaningful work is the subjective experience that one’s work has significance, facilitates personal growth, and contributes to the greater good (Steger et al., 2012). Scholars consider meaningful work to be a key outcome of self-determination, which itself is derived from having decent work (Duffy et al., in press b). Although self-determination may lead to more meaningful work, not all people have access to self-determined work (Blustein, 2001; Duffy et al., in press b). People who are perhaps the most constrained include people from lower social class backgrounds who do not have the privilege of occupational choice. People from lower social class backgrounds often lack access to societal resources (e.g., education) and may be coping with frequent economic insecurity, limiting the freedom to choose work that meets intrinsic needs, given the urgency of satisfying external needs (Blustein, 2001, 2013).

Therefore, in the current study, we broadly examined the notion that external factors restrict self-determination, which in turn predict the experience of meaningful work. Specifically, we tested whether social class and work volition (the perceived ability to choose one’s work) negatively predict internal regulation at work and, in turn, whether these variables positively predict meaningful work. We worked from two existing frameworks: (i) Self-Determination Theory (SDT; Ryan and Deci, 2002), which extensively outlines intrinsic and extrinsic human motivation and (ii) the Psychology of Working Framework (Blustein, 2001, 2013), which outlines the role of access to privilege and power in the satisfaction of work needs and overall work well-being.

### Self-Determination Theory

Self-Determination Theory is a macro theory of human motivation that describes and explains psychological needs, the continuum of external and internal regulation of behavior, and the attainment of psychological well-being (Ryan and Deci, 2002). Over the past forty years, SDT has been abundantly supported by empirical studies and has expanded to include applications of health (Ryan et al., 2008), work (Gagné and Deci, 2005), education (Black and Deci, 2000), relationships (Patrick et al., 2007), and psychotherapy (Ryan and Deci, 2008). It rests on the assumption that people display different types of motivation that are driven by three basic needs: autonomy, relatedness, and competence.

*Autonomy* refers to the extent to which one’s internal world is holistically integrated and self-regulated (Ryan and Deci, 2002). When people are acting autonomously, they have an internal locus of control and perceive their actions as self-directed. *Relatedness* refers to the extent to which one is cared for by and connected to others. When people’s relatedness needs are satisfied, they feel that they belong and have a sense of safety within their communities (Baumeister and Leary, 1995; Ryan and Deci, 2002). *Competence* refers to feeling a sense of mastery in one’s ability to interact with one’s environment as well as obtaining opportunities to express capacities on a regular basis (Ryan and Deci, 2002). When people’s competency needs are met, they feel confident in their abilities to navigate specific life domains and to control the outcomes of different activities.

According to SDT theory, psychological health and well-being are determined by the extent to which these three needs are satisfied and how much behavior is internally regulated. The extent to which one’s behavior is internally motivated is increased when psychological needs are met. Ryan and Deci (2008) described five types of motivation that range from intrinsic motivation, in which the individual engages in a given activity purely for personal fulfillment and enjoyment, to external regulation, in which one acts purely for external reward or to avoid a negative consequence. They also discuss amotivation, the absence of an intention to act. The types of motivation that fall between intrinsic and extrinsic motivation – introjected regulation, identified regulation, and integrated regulation – are forms of internal regulation that range in the extent to which behavior is self-directed. For introjected regulation, behavior is partially internalized but functions to avoid negative emotions and maintain self-esteem. In identified regulation, behavior is more autonomous and valued personally. In the most autonomous form of internal regulation, integrated regulation, regulations are assimilated into one’s self in accordance with one’s own values and needs. However, they are still partially external because behaviors are not done for their inherent enjoyment but, instead, for some other outcome.

Studies suggest that intrinsic motivation plays a key role in meaningful work and related constructs. One hypothesis is that engaging in intrinsically motivated work behavior creates congruence between work behaviors and one’s self-concept, which results in feelings of meaningfulness (Rosso et al., 2010). Despite the limited number of studies in this area, existing research supports this idea. For example, Steger et al. (2012) found significant correlations between intrinsic motivation and meaningful work, and Kashdan and Steger (2007) found that higher levels of state and trait curiosity at work led to greater perceptions of meaningfulness, more frequent growth-oriented behavior, and higher levels of life satisfaction. Recent literature on viewing one’s work as a calling (see Duffy and Dik, 2013 for a review) also provides evidence for a link between intrinsic motivation and experienced meaningfulness. A key-implied aspect of career calling is that it is intrinsically motivated work and satisfies the worker beyond external reward. Numerous studies have linked living a calling with increased life satisfaction, commitment to one’s career, life meaning, and meaningful work (Duffy and Dik, 2013). Taken together, the evidence provides support for a positive relation between intrinsic motivation and well-being in and outside of work.

### Psychology of Working Framework

Like SDT, Blustein’s (2001, 2013) PWF focuses on the satisfaction of universal human needs and their relation to well-being. Blustein (2001, 2013) proposed three groups of basic needs that have the potential to be satisfied through working: survival/power needs, relational needs, and self-determination needs. Unique to the PWF, however, is its core focus on the role of privilege and social power in satisfaction of needs and its encouragement of inclusivity and social justice in work research, policy, and practice. Scholars building upon the PWF have proposed the Psychology of Working Theory (PWT), which integrates notions of decent work (Duffy et al., in press b). Specifically, the PWT proposes that economic constraints and social marginalization impact access to decent work, work that is dignified, safe, and offers fair wages and social protection (International Labour Organization [ILO], 2015). Duffy et al. (in press b) further argue that decent work leads to self-determination, which in turn leads to meaningful and fulfilling work. Although many of these links have empirical support, others are yet to be tested, especially the links from self-determination to meaningful work.

From the perspectives of the PWF and the PWT, an important question is the extent to which workers have access to intrinsically motivated work. From the PWF lens, two primary factors that impact access to meaningful work are social class and work volition. Social class is a multidimensional construct that reflects a person’s status in society (Diemer and Ali, 2009). Recommended practices for assessment of social class include both objective and subjective measures (Diemer and Ali, 2009). Objective measures include income, education level, and occupational prestige – often referred to together as socioeconomic status (SES). In contrast, subjective social status (SSS) refers to people’s perceptions of their standing in society as compared to the general population and is obtained through self-report measures. Studies have found that SSS predicts psychological well-being above and beyond SES (Adler et al., 2008), which may make SSS a better choice when using psychological outcomes (Liu et al., 2004). Past studies have linked higher SES and SSS to greater levels of career commitment, meaningful work, and likelihood of living out a calling (Duffy et al., 2013; Allan et al., 2014).

A second key variable in access to intrinsically motivated work is work volition. Work volition refers to a person’s perception of freedom in work choice (Duffy et al., 2012). Work volition is an important construct that may be crucial for decent work. Specifically, inherent in the idea of decent work is that workers have the ability to choose work that is not exploitative or degrading and that offers greater opportunities for engaging in intrinsically motivating tasks. Although contextual variables – like social class and objective work barriers – relate to work volition, the correlations are weak; indicating that work volition is a person input distinct from social class (Allan et al., 2014). Previous research suggests that work volition is strong, positive predictor of meaningful work. For example, career calling studies show that work volition positively predicts a person’s likelihood to be living out their calling (Duffy and Autin, 2013; Duffy et al., in press a). Moreover, variance in work volition predicts living a calling above and beyond the effects of income and level of education (Duffy et al., in press b). The authors suggested that perceptions of constraints in work choice may impede people from obtaining intrinsically rewarding work.

Only one known study has examined both social class and work volition in predicting meaningful work. In a two-part study, Allan et al. (2014) examined (i) sources of meaningful work and (ii) levels of meaningful work in people from various social class backgrounds. They found that, although people from higher social class backgrounds were more likely to endorse meaningful experiences at work, people from across social class backgrounds endorsed similar sources of meaningful work. In particular, prosocial impact was reported by the vast majority of participants when asked what made their jobs meaningful, regardless of social class background. This suggests that the underlying mechanisms of meaningful work may be similar across class backgrounds; however, it appears that being from a lower class background is associated with barriers to meaningful work.

Although Allan et al. (2014) laid a foundation for examining social class and meaningful work, their study had several limitations. First, the study was limited in its measurement of social class. Specifically, the authors used only SES and a categorical measure of SSS, despite best practice recommendations to use continuous measures of SSS (Diemer and Ali, 2009). Second, although the authors referred to SDT and implicitly incorporated SDT assumptions, they did not directly test motivational variables. Therefore, in the current study, we aim to build on Allan et al. (2014) by (i) using a more sophisticated measure of social class and (ii) testing the extent to which SDT motivation variables predict meaningful work.

### The Present Study

The overall goal of this study was to better understand how SDT and PWF variables predict meaningful work. Building off previous research (Blustein, 2001, 2013; Ryan and Deci, 2002; Steger et al., 2012), we sampled a large and diverse group of employed adults and proposed a structural model with social class and work volition predicting SDT motivation variables and with these variables predicting meaningful work. Specifically, we predicted that social class and work volition would both show strong positive relations to internal regulation and strong negative relations to extrinsic motivation and amotivation. In turn, we hypothesized that internal regulation would predict greater meaningful work but that amotivation and extrinsic motivation would predict less meaningful work. We also hypothesized there would be indirect effects from work volition and social class to meaningful work via the motivation variables. Reflecting our hypotheses above, we predicted that indirect effects through amotivation and extrinsic motivation would be negative and that the indirect effects through the internal regulation would be positive.

To investigate the viability of this model we also tested two alternative models. In the first alternative model, we tested a model similar to Allan et al. (2014). This study found work volition to mediate the relation between social class and meaningful work, suggesting that social class could potentially be a predictor, rather than a correlate, of work volition. In the second alternative model we tested a different permutation of the indirect effects. Given that our hypotheses were based on cross-sectional data, meaningful work could be positioned before work motivation. Specifically, work may become perceived as internally regulated when people perceive it as meaningful. Therefore, we tested an alternative model with meaningful work mediating the relation between social class and work volition and the six motivation variables.

## Materials and Methods

### Participants

The sample consisted of 339 working adults living in the United States. Participants ranged in age from 18 to 71 (*M* = 35.33 and *SD* = 11.78) and self-identified as female (*N* = 187, 55.2%), male (*N* = 146, 43.1%), transgender (*N* = 2, 0.6%), and other (*N* = 3, 0.9%). In terms of race/ethnicity, participants were able to select multiple answers. Participants mainly self-identified as White/European American/Caucasian (*N* = 272, 80.2%), with remaining participants identifying as African/African-American (*N* = 27, 8.0%), Asian/Asian American (*N* = 22, 6.5%), Hispanic/Latinao American (*N* = 19, 5.6%), American Indian/Native American (*N* = 9, 2.7%), Asian Indian (*N* = 6, 1.8%), Arab American/Middle Eastern (*N* = 1, 0.3%), and Other (*N* = 2, 0.6%). In terms of education, 11.8% (*N* = 40) had a high school education or less, 4.5% (*N* = 15) had a trade or vocational school diploma, 33.0% (*N* = 112) had some college, 36.9% (*N* = 125) had a college degree, and 13.0% (*N* = 44) had a professional or graduate degree. The sample captured a wide range of occupations with 248 unique job titles represented. The most frequently reported job titles included customer service representative (3.5%; *N* = 12), administrative assistant (3.2%; *N* = 11), sales representative (3.2%; *N* = 11), computer programmer (1.8%; *N* = 6), teacher (1.8%; *N* = 6), writer (1.8%%; *N* = 6), and office manager (1.5%, *N* = 6).

### Instruments

#### Social Class

Social class was measured with the MacArthur Scale of subjective social status (Adler et al., 2000). Participants are given a picture of a ladder and the following instructions: “Think of this ladder as representing where people stand in our society. At the top of the ladder are the people who are the best off, those who have the most money, most education, and best jobs. At the bottom are the people who are the worst off, those who have me least money, least education, and worst jobs or no jobs.” Participants are then asked to indicate where they fall on the ladder on a scale from 1* = bottom rung* to 10* = top rung*. Adler et al. (2000) found the measure to relate to measures of psychological functioning and health-related factors (e.g., heart rate), and most relations remained significant after controlling for objective social status (e.g., income, education, etc.). Other studies have found scores on the measure to significantly and positively correlate with level of employment, education, income, wealth, standard of living, and perceptions of financial security (Adler et al., 2008).

#### Meaningful Work

The degree to which participants felt their work was meaningful was measured with the 10-item Work as Meaning Inventory (WAMI; Steger et al., 2012). Steger et al. (2012) found the scale to load onto three factors (i.e., positive meaning, meaning-making through work, and greater good motivations) that loaded onto a higher order meaningful work factor. Sample items include “I have found a meaningful career,” and “The work I do serves a greater purpose.” Participants answered items on a 7-point scale ranging from 1 (*strongly disagree*) to 7 (*strongly agree*). Points from each item were summed to calculate a total score, with higher scores representing higher levels of meaningful work. In the instrument development study, Steger et al. (2012) found the scale to correlate in the expected direction with overlapping variables, such as career commitment, presence of life meaning, job satisfaction, and calling. Furthermore, Steger et al. (2012) found the WAMI to have high internal consistency (α = 0.93), and in the present study, the estimated internal consistency was α = 0.94.

#### Work Volition

Work volition was measured with the Work Volition Scale (WVS; Duffy et al., 2012). Duffy et al. (2012) found items from the WVS to load onto three factors (i.e., volition, financial constraints, and structural constraints) that, in turn, loaded onto a higher order work volition factor. The scale includes 13 items on a 7-point Likert scale ranging from 1 (*strongly disagree*) to 7 (*strongly agree*). Sample items include, “I feel total control over my job choices,” “Due to my financial situation, I need to take any job I can find” (reverse coded), and “Negative factors outside my personal control had a large impact on my current career choice” (reverse coded). Higher scores are associated with higher work volition. Duffy et al. (2012) found the WVS to correlate in the expected directions with work locus of control, job satisfaction, discrimination, and career barriers. They also reported an estimated internal consistency of α = 0.86. The estimated internal consistency for the present study was α = 0.91.

#### Work Motivation

Work motivation was assessed with the Work Extrinsic and Intrinsic Motivation Scale (WEIMS; Tremblay et al., 2009). The scale consists of 18 items on a 7-point Likert-type scale ranging from 1 (*does not correspond at all*) to 7 (*corresponds exactly*). The measure includes six subscales, each containing three items, corresponding to Ryan and Deci’s (2000) increasing levels of self-determination. Tremblay et al. (2009) found scores on the scale to load predictably on the six factors and to correlate in the expected directions with one another and work-related outcomes, such as job satisfaction, organizational commitment, and turnover intentions. The authors also reported estimated internal consistencies of α = 0.80 (intrinsic motivation), α = 0.83 (integrated regulation), α = 0.67 (identified regulation), α = 0.70 (introjected regulation), α = 0.77 (external regulation), and α = 0.64 (amotivation), acceptable internal consistencies given that the scales only consisted of three items each. The estimated internal consistencies for the six subscales in the present study were α = 0.89 (intrinsic motivation), α = 0.89 (integrated regulation), α = 0.76 (identified regulation), α = 0.75 (introjected regulation), α = 0.69 (external regulation), and α = 0.83 (amotivation).

### Procedure

Participants joined the study through Mechanical Turk (MTurk). MTurk is an online participant source that allows people to complete surveys for monetary compensation, although most respondents report completing surveys for enjoyment (Buhrmester et al., 2011). Recent reviews and studies examining MTurk have largely concluded that it produces valid data that is comparable to laboratory and other internet recruitment methods (Buhrmester et al., 2011; Sprouse, 2011). A link including an informed consent document and the survey itself was posted on MTurk, and in order to participate, participants had to (i) be over the age of 18, (ii) reside within the United States, and (iii) be employed at least part-time. Participants were given $0.25 for completing the survey completed, which is consistent with typical amounts offered to participants on MTurk.

The initial sample included 430 participants. However, 22 were unemployed and therefore did not meet inclusion criteria, and 21 people only answered demographic questions. Additionally, 36 participants did not respond correctly to three validity items. Finally, 12 cases only completed the first two questionnaires and were, therefore, missing data on seven or more study variables. All these cases were removed, leaving a final sample size of 339. Of this sample, 283 (83.5%) participants had complete data, 49 (14.5%) were missing data on one study variable, and 7 (2.1%) were missing data on two study variables. For the remaining missing data, we used Full Information Maximum Likelihood (FIML) to impute values for missing data (Tabachnick and Fidell, 2007). FIML uses all available information to calculate estimates with added error so as to not bias estimates. Experts (Tabachnick and Fidell, 2007) argue that approaches like FIML are superior to the traditional techniques, such as list-wise deletion and mean substitution, which tend to discard valuable information and bias estimates.

### Data Analysis

To test the hypotheses discussed above, we used structural equation modeling in AMOS 22. We first conducted preliminary analyses to evaluate for non-normality and the existence of outliers and to obtain correlations among study variables. We then tested a measurement model to evaluate if all indicators loaded onto their respective factors with good fit, then moved onto testing the structural model. To assess model fit, we used indices that minimized the likelihood of Type I and Type II error (Hu and Bentler, 1999). These were the chi-square test (χ^2^), the comparative fit index (CFI), and the root mean square error of approximation (RMSEA). A significant χ^2^ can indicate a poor fitting model, but this test is not reliable in larger samples (Tabachnick and Fidell, 2007). Criteria for the CFI and RMSEA have ranged from less conservative (CFI ≥ 0.90 and RMSEA ≤ 0.10) to more conservative (CFI ≥ 0.95 and RMSEA ≤ 0.08; Hu and Bentler, 1999; Weston and Gore, 2006). However, criteria should not be used as strict cut-offs, and researchers should consider sample size and model complexity when judging the fit of models (Weston and Gore, 2006).

After testing the structural model, we calculated its indirect effects. Because AMOS 22 only gives significance tests for the combined indirect effects (i.e., the effect of work volition on meaningful work through all three work motivation variables), we used RMediation (Tofighi and MacKinnon, 2011) to produce confidence interval estimates tests for individual indirect effects. These indirect effects are significant when they do not include zero. Finally, we evaluated the fit of our alternative models and compared their fit to the structural model.

## Results

### Preliminary Analyses

No variables had scores above 3.25 standard deviations from the mean, so no cases were removed due to outliers. All study variables had absolute values of skewness and kurtosis less than one, and all except for amotivation appeared normally distributed on visually inspected histograms and boxplots. Amotivation appeared positively skewed due to many low scores on the scale. However, given that its absolute value of skewness was under one (0.85) and that amotivation scores fell relatively normally other than the floor effect, we did not transform the variable.

We also ran preliminary correlations on the manifest study variables. The four variables representing internally motivated behavior were highly correlated (*r* = 0.73–0.77). This raised issues of multicollinearity and the potential for the variables to represent a single factor. This is consistent with Tremblay et al.’s (2009) suggestions of summing subscales into self-determined (intrinsic motivation, integrated regulation, and identified regulation) and non-self-determined (introjected, external, and amotivation) subscales. The authors found these subscales to predict work-related variables, such as organizational commitment, in the expected directions, and scholars have used these subscales in subsequent studies (e.g., Shu, 2015). To explore this further, we ran an exploratory factor analysis on all WEIMS items. We used principal axis factoring with promax rotation based on Eigenvalues greater than one (Tabachnick and Fidell, 2007). The items loaded on three factors at values of 0.51 or above. The first factor included all items from the internal regulation variables (i.e., intrinsic motivation, integrated regulation, identified regulation, and introjected regulation), the second factor included the external regulation items, and the third factor included the amotivation items.

Therefore, similar to the recommendation made by Tremblay et al. (2009) we measured a single internal regulation variable (intrinsic motivation, integrated regulation, and identified regulation). However, given the results of the exploratory factor analysis above, we loaded introjected regulation onto the internal regulation variable. First, the three items form this subscale had high factor loadings from the exploratory factor analysis (0.62, 0.75, and 0.53). Second, introjected regulation represents behaviors undertaken to regulate self-esteem based on external factors. However, it is partially internalized (Ryan and Deci, 2002) and could reasonably be grouped with other internally regulated variables. Regardless, only its shared variance with the self-determined variables would be included in the latent factor.

**FIGURE 1:**
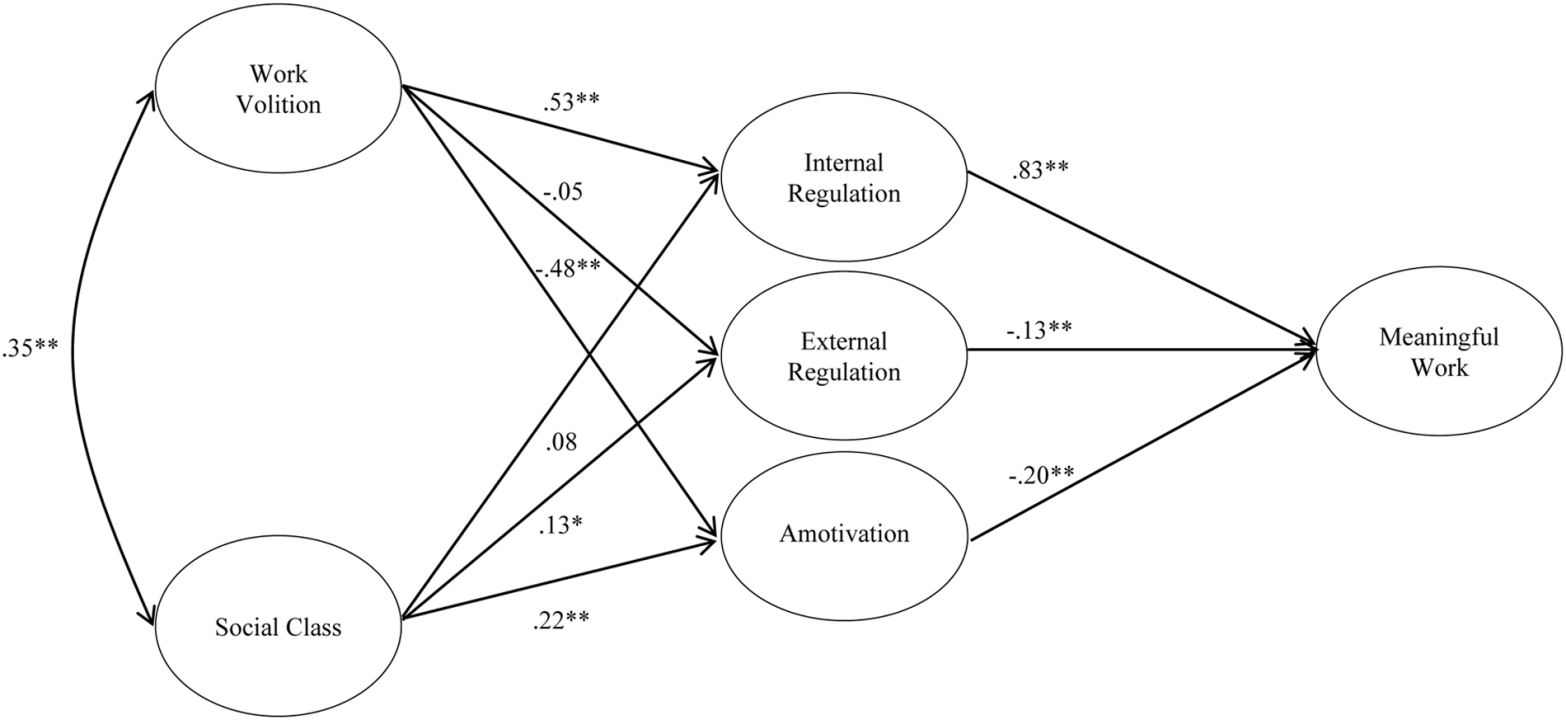
**Final structural model with standardized regression coefficients.** Correlations among work motivation variables and errors are not depicted in the figure (^∗^*p* < 0.05 and ^∗∗^*p* < 0.01).

Also contrary to Tremblay et al. (2009), we did not load introjected regulation, external regulation, and amotivation onto a single factor. In our sample, amotivation was unrelated to introjected regulation (*r* = 0.10 and *p* = 0.08) and external regulation (*r* = 0.00 and *p* = 1.00), and external regulation had only a small correlation with introjected regulation (*r* = 0.12, *p* < 0.05). Although these correlations are consistent with Tremblay et al.’s (2009) results, they reveal that these variables clearly do not represent a single construct, especially when viewed in light of the exploratory factor analysis described above. Therefore, we kept them separate in the structural model (**Figure 1**).

### Measurement Model

Before testing the structural mediation model, we tested a measurement model with all study variables. The three subscales for work volition and meaningful work and the individual items for external regulation and amotivation were loaded onto their respective factors. The sum of the subscales for intrinsic motivation, integrated regulation, identified regulation, and introjected regulation were loaded onto an internal regulation latent variable. Because social class was a single item, it was included as a manifest variable. All variables were allowed to correlate. This model was an acceptable fit to the data, χ^2^(105) = 369.47, *p* < 0.001, CFI = 0.92, and RMSEA = 0.09, 95% CI [0.8, -0.10], and all indicators loaded on their factors with coefficients of 0.44 or higher. **Table 1** depicts the factor correlations among study variables. Meaningful work was significantly and positively related to work volition (0.57), social class (0.22), and internal regulation (0.84). Meaningful work was also negatively related to external regulation (-0.16) and amotivation (-0.25).

**Table 1:** Descriptive statistics and factor correlations of study variables.

	1	2	3	4	5	6
1. Work volition	–					
2. Social class	0.35^∗∗^	–				
3. Meaningful work	0.57^∗∗^	0.22^∗∗^	–			
4. Internal regulation	0.55^∗∗^	0.26^∗∗^	0.84^∗∗^	–		
5. External regulation	-0.001	0.11	-0.16^∗∗^	-0.04	–	
6. Amotivation	-0.40^∗∗^	0.05	-0.25^∗∗^	-0.06	0.03	–
Mean	55.36	4.98	46.78	51.54	15.20	7.42
Standard deviation	16.99	1.64	14.49	16.80	3.83	4.40


*^∗^*p* < 0.05; ^∗∗^*p* < 0.01.*

### Structural Model

The structural model included work volition and social class predicting meaningful work via the work motivation variables. As with the measurement model, all indicators loaded on their latent factors, except for social class, which was represented as a manifest variable. We tested both partial and full mediation models. The partial mediation model included direct paths from work volition and social class to meaningful work. This model had good fit to the data, χ^2^(105) = 369.47, *p* < 0.001, CFI = 0.92, and RMSEA = 0.09, 95% CI [0.8, -0.10]. The full mediation model did not include direct paths from social class and work volition to meaningful work, and it also had acceptable fit, χ^2^(107) = 371.49, *p* < 0.001, CFI = 0.92, and RMSEA = 0.09, 95% CI [0.8, -0.10]. Given that the difference in chi-square between models was not significant, χ^2^(2) = 2.02, *p* = 0.64, we retained the full mediation model for its parsimony. **Figure 1** displays the final, structural model with standardized regression coefficients. This model explained 77% of the variance in meaningful work.

### Indirect Effects

We tested each indirect effect from work volition and social class to meaningful work via the three work motivation variables. The indirect effects from work volition to meaningful work via internal regulation (c’ = 0.60, SE = 0.08, 95% CI = 0.44, 0.76) and amotivation (c’ = 0.13, SE = 0.03, and 95% CI = 0.07, 0.20) were significant but the indirect effect via external regulation (c’ = 0.01, SE = 0.01, and 95% CI = -0.02, 0.04) was not. The indirect effects from social class to meaningful work via internal regulation (c’ = 0.25, SE = 0.16, and CI = -0.08, 0.57) was not significant, but the indirect effects via external regulation (c’ = -0.06, SE = 0.04, and CI = -0.14, -0.001) and amotivation (c’ = -0.16, SE = 0.05, and CI = -0.27, -0.06) were significant.

### Alternative Models

First, we tested an alternative model following from the model described by Allan et al. (2014). In this model, social class directly predicted work volition but did not predict the work motivation variables. This model had comparable fit to the structural model, χ^2^ (110) = 390.21, *p* < 0.001, CFI = 0.92, and RMSEA = 0.09, 95% CI [0.8, -0.10], but it had a significantly greater chi-square, χ^2^ (3) = 18.72 and *p* < 0.001, indicating that the fit of the structural model was better. As described above, we also tested another alternative model that replaced meaningful work with work motivation as the outcome variable. In this model work volition and social class predicted meaningful work, which in turn predicted internal regulation, external regulation, and amotivation. This model had significantly worse fit than the structural model, χ^2^ (111) = 424.23, *p* < 0.001, CFI = 0.91, and RMSEA = 0.09, 95% CI [0.8, -0.10]; χ^2^ (4) = 52.74 and *p* < 0.001. Because neither alternative model significantly improved the fit of the model or the understanding among the study variables, we retained the structural model.

## Discussion

The goal of the current study was to advance the literature on meaningful work by examining how core variables within SDT (Ryan and Deci, 2002) and the Psychology of Working Framework (Blustein, 2013) predict meaningful work and, in turn, the degree to which these variables are predicted by socioeconomic constraints. Both social class and work volition predicted amotivation whereas only work volition predicted internal regulation and only social class predicted external motivation. All three types of work motivation significantly predicted meaningful work. However, when all three variables were included in the same model, internal motivation emerged as the largest predictor. The strength of this path coefficient provides initial evidence that being internally motivated at work may be essential to experiencing meaningful work.

In the current paper internal regulation was represented by intrinsic motivation, introjected regulation, identified regulation, and integrated regulation. All of these motivation styles refer to being internally motivated but differ with regards to the degree of self-direction amongst behaviors (Ryan and Deci, 2002). Despite this difference our factor analysis demonstrated these four styles were best represented by one underlying internal regulation construct. The overlap of internal regulation and meaningful work was so high that it could imply a construct overlap. However, when examining the internal regulation instrument items, it is clear that these are less about meaning and more about viewing work as satisfying, enjoyable, and connected to one’s present and future self. When work is approached in this way, our findings indicate that, as hypothesized, there is an extremely high likelihood of experiencing meaningful work. Although previous studies have found strong correlates of meaningful work (e.g., living a calling, career commitment, person environment fit; Duffy et al., 2013, 2015), none have approached the level found in the current study with internal regulation, suggesting it may be an necessary predictor variable.

Also supporting hypotheses, external motivation and amotivation were each negatively related to meaningful work, even when accounting for the high amount of variance contributed by internal motivation. Although the strength of these path estimates were small, they are still important to consider, because people who were extrinsically motivated to work (e.g. for income, security) or who had a lack of motivation (e.g., unsure why they are working at all) were less likely to experience their work as meaningful. This latter motivation category is especially important to consider when assessing the role of socioeconomic constraints in the overall model.

Our findings indicated that although neither work volition nor social class directly predicted meaningful work, their indirect effects were evident through internal regulation, external regulation, and amotivation. As hypothesized, work volition was a strong, positive predictor of internal regulation and a strong, negative predictor of amotivation. Specifically, people who felt more in control of their career decision making were more likely to have high levels of internal regulation and low levels of amotivation, which in turn predicted meaningful work. These findings demonstrate the freedom of choice-motivation link, which is an underlying principle of SDT. When individuals feel autonomy and choice in a certain life domain they will be more likely to feel motivated and engaged in that domain, resulting a positive appraisal of that domain (e.g., meaning, satisfaction, and persistence; Ryan and Deci, 2002). Importantly, the converse is also true: people with little choice in their careers will likely feel lower levels of internal regulation, higher levels of amotivation, and in turn less meaningful work.

Finally, there were small but significant positive links from social class to amotivation and external regulation. Bivariate relations among these variables were not significant, and only in the full model do these paths become significant, implying that greater social class is linked with greater external regulation and amotivation. This counterintuitive finding implies that people from higher social class backgrounds are more likely to demonstrate an attention to extrinsic rewards and a lack motivation toward work. However, this was evident only when work volition was included alongside social class in the model, which likely suppressed the relations from social class to external regulation and amotivation. In other words, those from higher social class backgrounds were more likely to be externally regulated and amotivated once their degree of work volition was accounted for. It is possible that without the higher work volition associated with higher social classes, people are more vulnerable to external regulation (e.g., working for money) and amotivation. This may represent findings from a small group of people in higher social classes who are privileged but feel stuck in their jobs. Although this is speculative, future studies may wish to further investigate this surprising result.

### Limitations and Future Directions

The results and conclusions from this study need to be considered in light of a number of limitations, each of which offer directions for future research. First and foremost, the data gathered for this study were cross-sectional, and we were unable to make causal assertions of how these variables affect one another over time. For example, a longitudinal study could provide more information about whether or not social class and work volition are best positioned as correlates or if work volition is an outcome of social class over time (Allan et al., 2014). Additionally, it may be that meaningful work predicts or has recursive effects on work motivation, another area that could be examined with longitudinal data. Second, data for this study were collected only from US participants. Although large-scale studies have documented how SDT works cross culturally (Church et al., 2013), it is necessary to understand if the setup of variables in this study’s model also hold across cultures.

Third, this study contains SDT variables related to motivation but does not include variables related to need satisfaction, such as autonomy, relatedness, and competence. These are theoretically proposed to proceed motivation (Ryan and Deci, 2002), and it would be important to test a more complete model which includes these variables as potential mediators connecting aspects of social class to work motivation. Fourth, future research in this area might expand outcomes to include variables related to overall well-being. For example, it would be relevant to understand how meeting needs at work predicts life meaning and satisfaction in addition to meaningful work. Doing so would allow for a more complete picture of predictors and outcomes of SDT constructs in the work domain. Fifth, it will be important for future researchers to connect results from the current study to other variables within the PWF. Specifically, there is little empirical data on decent work. The construct of decent work represents a much of the real world applicability of research on work and life meaning, given the lack of access to decent work for many people. Future researchers might strengthen the knowledge base in this area by linking decent work to SDT motivation and need satisfaction variables.

A final limitation of this study illustrates the PWF’s criticism of vocational research in general (Blustein, 2001). Our sample was largely White, had more income than average, and was highly educated. This is partially a problem with online data collection in that it reflects disparities in who uses and has most access to the internet (Etter and Perneger, 2001; Rhodes et al., 2003) and could bias our results in favor of people with relatively more power and privilege. Therefore, our results should be tested and replicated with samples that proportionally reflect different groups in the United States. Relatedly, future studies should actively recruit members of diverse groups, whether participants are being recruited online or from the community.

## Author Contributions

BA and KA conceptualized the study, chose the theoretical framework, chose measures, designed the questionnaire, and collected the data. BA analyzed the data and wrote the methods and results. KA wrote the introduction. RD read the paper, provided suggestions for revisions, and wrote the discussion after revisions were made. All authors read and revised the manuscript several times.

## Conflict of Interest Statement

The authors declare that the research was conducted in the absence of any commercial or financial relationships that could be construed as a potential conflict of interest.

The reviewer Letizia Palazzeschi and handling Editor declared their shared affiliation, and the handling Editor states that the process nevertheless met the standards of a fair and objective review.
